# Supplementary material for: The C4 Protein of TbLCYnV Promotes SnRK1 β2 Degradation Via the Autophagy Pathway to Enhance Viral Infection in N. benthamiana
Source: Viruses. 2024 Feb 1;16(2):234. doi: 10.3390/v16020234 (PMC10892878; doi:10.3390/v16020234)
Supplement: Supplementary file 1 [file viruses-16-00234-s001.zip › viruses-2747436-Supplementary-Materials.pdf]

Supplementary Materials

Table S1. Primers used in this study.

Table S1. Primers used in this study.

**Name**	**Sequence (5’-3’)**
BD- C4-F	ATGGCCATGGAGGCCGAATTCATGGGAGCCCTCATCTCC
BD- C4-R	CCGCTGCAGGTCGACGGATCCTTACATTAAGAGCCTCTGACTTACTG
AD-NbSnRK1 β2-F	ATGGCCATGGAGGCCAGTATGGGGAATGTTAATGGAAGAGAA
AD-NbSnRK1 β2-R	GCAGCTCGAGCTCGATCACCTCTGTATTGACTTGTAAAGAACC
C4-mCherry-F	GACGAGCTGTACAAGATGGGAGCCCTCATCTCC
C4-mCherry-R	AACGAGCTCTGTCGATTACATTAAGAGCCTCTGACTTACTGC
NbSnRK1β2-GFP-F	GAGCTCGGTACCCGGGGATCCATGGGGAATGTTAATGGAAGAGAAG
NbSnRK1β2-GFP-R	GCCCTTGCTCACCATGTCGACCCTCTGTATGGACTTGTAAAGTACC
2YN-C4-F	ATTTACGAACGATAGATGGGAGCCCTCATCTCC
2YN- C4-R	ACCTCCTCCACTAGTCATTAAGAGCCTCTGACTTACTGC
2YC-NbSnRK1 β2-F	ATTTACGAACGATAGATGGGGAATGTTAATGGAAGAGAAG
2YC-NbSnRK1 β2-R	ACCTCCTCCACTAGTCCTCTGTATGGACTTGTAAAGTACC
TRV-NbSnRK1 β2-F	GCGGATCCGGATGTGAAGGATTATGTTCCAGA
TRV-NbSnRK1 β2-R	GCCTCGAGGTGGAGGTCTCGAGAGAGG
NbSnRK1 β2-RT-qPCR-F	AAGGTGGCTCCTTGGCATAG
NbSnRK1 β2-RT-qPCR-R	TGGAGGTGTTCCCCTGACTT
ACTIN-F	CAATCCAGACACTGTACTTTCTCTC
ACTIN-R	AAGCTGCAGGTATCCATGAGACT
TbLCYnV qPCR-F	ATGTCCCTAGAGGATGTGAAGG
TbLCYnV qPCR-R	CTATCCAGAATAAAACAGTGTTAGTGTGG
V1-F	ATGTCGAAGCGACCAGC
V1-R	TTAATTCGTCACTGAGTCATAAAAATAACTCC
C4-X1-F	ATGGCCATGGAGGCCGAATTCATGTCGAAGGGAAATACGTCTGCA
C4-X1-10-R	GCCGCTGCAGGTCGACTTACATTAAGAGCCTCTGACTTACTGC
C4-X2-10-1-F	ATGGCCATGGAGGCCGAATTC
C4-X2-1-R	ACTGGATAAGCACGTGGAG
C4-X2-2-F	ACGTGCTTATCCAGTGATTCTTCGACTTGGTATCCCC
C4-X3-1-R	TGTTATTCGTGCAGACGTATTTCC
C4-X3-2-F	TCTGCACGAATAACACAGCACATTTCCATCCGAAC
C4-X4-1-R	ACCTGGTTGGGGATACC
C4-X4-2-F	TATCCCCAACCAGGTCTAAATCCAGCTCCGGCG
C4-X5-1-R	CTCCCTGAATGTTCGGATGG
C4-X5-2-F	CGAACATTCAGGGAGTCGACAAGGACGGAGACAC
C4-X6-1-R	TGTAGGACTTGACGCCGG
C4-X6-2-F	GCGTCAAGTCCTACAGAGAGTTTCAGATCGACGGAAG
C4-X7-1-R	CCCATTCGAGGGTGTCTC
C4-X7-2-F	ACACCCTCGAATGGGGAGGGGGTCAACAGACAG

C4-X8-1-R	TTGCAGATCTTCCGTCGATC
C4-X8-2-F	ACGGAAGATCTGCAAACGCCCAGGCACTTAAC
C4-X9-R	GCCGCTGCAGGTCGACTTAAAGCGTCATTGGCTGTCTG
C4-X10-R	ATGGCCATGGAGGCCGAATTCATGGGAGCCCTCATCTCC
C4(D22A)-F	GAATAACAGCATCTTCGACTTGGTA
C4(D22A)-R	GTCGAAGATGCTGTTATTCGTG
C4(S23A)-F	GAATAACAGATGCATCGACTTGG
C4(S23A)-R	GTCGATGCATCTGTTATTCGTGC
C4(S24A)-F	CAGATTCTGCAACTTGGTATCCC
C4(S24A)-R	CCAAGTTGCAGAATCTGTTATTCG
C4(T25A)-F	GATTCTTCGGCATGGTATCCC
C4(T25A)-R	GGATACCATGCCGAAGAATCTG
C4(W26A)-F	CTTCGACTGCATATCCCCAAC
C4(W26A)-R	GGGATATGCAGTCGAAGAATCTG
C4(Y27A)-F	CGACTTGGGCACCCC
C4(Y27A)-R	GGGGTGCCCAAGTCG
C4(P28A)-F	GACTTGGTATGCACAACAAGGT
C4(P28A)-R	GTTGTGCATACCAAGTCGAAG
C4(Q29A)-F	CCCGCACAAGGTCAG
C4(Q29A)-R	CCTTGTGCGGGATACCAAG
C4(P30A)-F	CCCCAAGCAGGTCAGC
C4(P30A)-R	CCTGCTTGGGGATACCAAG
C4(G31A)-F	CCCCAACAAGCACAGC
C4(G31A)-R	GCTGTGCTTGTTGGGG
pGEM-TbLCYnV-1A- F	GGCATGTACTCATGCCTCT
pGEM-TbLCYnV-1A-R	CATTTGGAGACACCTATATATTGTCTCCTG
pGEM-TbLCYnV-0.5A- F	CAGGAGACAATATATAGGTGTCTCC
pGEM-TbLCYnV-0.5A-R	AGAGGCATGAGTACATGCC
Pbinplus-TbLCYnV-0.5A- F	ATGCCTGCAGGTCGACTCTAGACAGGAGACAATATATAGGTGTCTCC
Pbinplus-TbLCYnV-0.5A- R	GCATACACAGGATTAGAGGCATGAGTACATGCCATATAC
NbSnRK1α-F	ACAATCGCCATCGTGTTTCC
NbSnRK1α-R	TGGAGTCCAAGAGCCCATTTC
AD-NbSnRK1 α-F	ATGGCCATGGAGGCCACAATCGCCATCGTGTTTCC
AD-NbSnRK1 α-R	GCAGCTCGAGCTCTGGAGTCCAAGAGCCCATTTC
BD-NbSnRK1 β2-F	ATGGCCATGGAGGCCATGGGGAATGTTAATGGAAGAGAAG
BD-NbSnRK1 β2-R	CCGCTGCAGGTCGACTCACCTCTGTATGGACTTGTAAAGTACC

Table S2. Sequences used for phylogenetic trees.

Table S2. Sequences used for phylogenetic trees.

Name	Accession numbers
Capsicum annuum SnRK1 β2	*XM_016695113.2*
Capsicum annuum SnRK1 β2-2	*XM_016698668.2*

Coffea arabica SnRK1 β2	*XM_027230380.1*
Coffea arabica SnRK1 β2-2	*XM_027233736.1*
Coffea eugenioides SnRK1 β2	*XM_027329704.1*
Ipomoea triloba SnRK1 β2	*XM_031246400.1*
Ipomoea nil SnRK1 β2	*XM_019328376.1*
Lycopersicon esculentum SnRK1 β2	*AF322108.1*
Nicotiana attenuata SnRK1 β2	*XM_019367914.1*
Nicotiana attenuata SnRK1 β2-2	*XM_019389930.1*
Nicotiana sylvestris SnRK1 β2	*XM_009775324.1*
Nicotiana sylvestris SnRK1 β2-2	*XM_009782492.1*
Nicotiana sylvestris SnRK1 β2-3	*XM_009782493.1*
Nicotiana tabacum SnRK1 β2	*XM_016584004.1*
Nicotiana tabacum SnRK1 β2-2	*XM_016660579.1*
Nicotiana tabacum SnRK1 β2-3	*XM_016634309.1*
Nicotiana tabacum SnRK1 β2-4	*XM_016595865.1*
Nicotiana tomentosiformis SnRK1 β2	*XM_009591992.3*
Nicotiana tomentosiformis SnRK1 β2-2	*XM_009621896.3*
Salvia hispanica SnRK1 β2	*XM_048087909.1*
Solanum lycopersicum SnRK1 PtoR Sip1	*AK320167.1*
Solanum lycopersicum SnRK1 PtoR Sip1-2	*NM_001247609.2*
Solanum lycopersicum SnRK1 PtoR Sip1-3	*XM_010322989.3*
Solanum lycopersicum Tau2	*NM_001279247.1*
Solanum pennellii SnRK1 β2	*XM_015201678.2*
Solanum pennellii SnRK1 β2-2	*XM_015219111.2*
Solanum pennellii SnRK1 β2-3	*XM_015219112.2*
Solanum stenotomum SnRK1 β2	*XM_049523959.1*
Solanum tuberosum SnRK1 β2	*XM_006352261.2*
Solanum tuberosum SnRK1 β2-2	*XM_006365779.2*
Solanum verrucosum SnRK1 β2	*XM_049497406.1*
Solanum verrucosum SnRK1 β2-2	*XR_007442465.1*

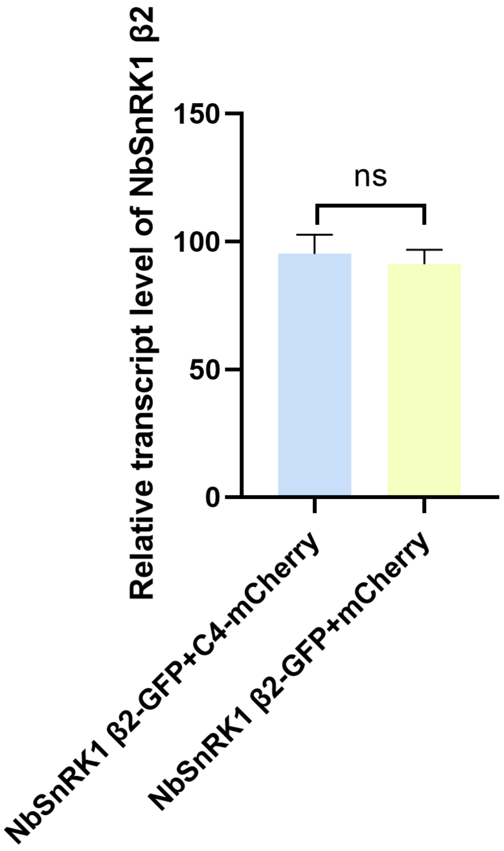

Figure S1. qRT-PCR analysis showing the *NbSnRK1 β2* transcript levels in *N. benthamiana* leaves co-expressed NbSnRK1 β2-GFP with C4-mCherry or mCherry. ns indicates no significant difference between the two treatments according to Student's t-test.

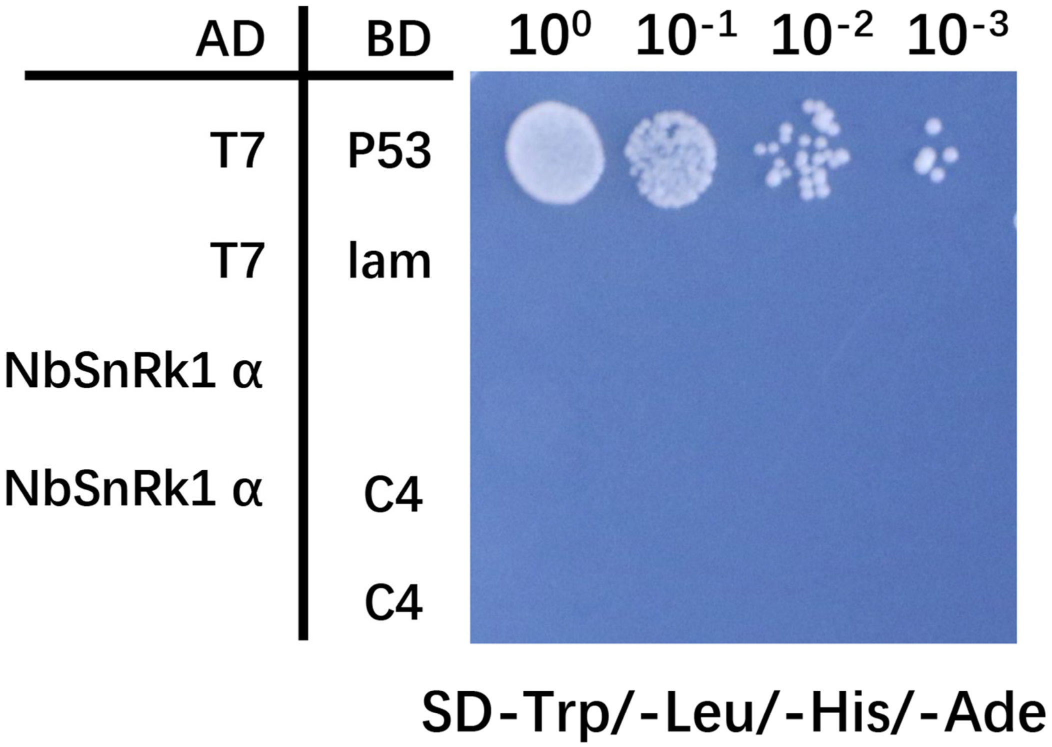

Figure S2. Exploring the interaction between TbLCYnV C4 and NbSnRK1 α by Y2H assay.
